# The Effect of Irrigation Fluid on Periprosthetic Joint Infection in Total Hip and Knee Arthroplasty: A Systematic Review and Meta-Analysis

**DOI:** 10.7759/cureus.7813

**Published:** 2020-04-24

**Authors:** Thomas Wood, Seper Ekhtiari, Raman Mundi, Mustafa Citak, Parag K Sancheti, Ernesto Guerra-Farfan, Emil Schemitsch, Mohit Bhandari

**Affiliations:** 1 Orthopaedic Surgery, McMaster University, Hamilton, CAN; 2 Surgery, McMaster University, Hamilton, CAN; 3 Orthopaedic Surgery, Helios Endo-Klinik, Hamburg, DEU; 4 Orthopaedics, Sancheti Institute for Orthopaedics and Rehabilitation, Pune, IND; 5 Orthopaedic Surgery, Vall D'hebron University Hospital, Barcelona, ESP; 6 Orthopaedic Surgery, Western University, London, CAN

**Keywords:** total joint arthroplasty, total knee arthroplasty, total hip arthroplasty, total knee replacement, total hip replacement, periprosthetic joint infection, wound lavage, irrigation, betadine, chlorhexidine

## Abstract

Introduction

Rates of osteoarthritis and total joint arthroplasty (TJA) are on the rise globally. Periprosthetic joint infection (PJI) is the most devastating complication of TJA. A number of different intraoperative interventions have been proposed in an effort to reduce infection rates, including antibiotic cements, local antibiotic powder, and various irrigation solutions. The evidence on the importance of irrigation solutions is limited but has gained prominence recently, including the publication of a large randomized controlled trial (RCT). Thus, the purpose of this study was to evaluate the effectiveness of various irrigation solutions and pressures at reducing the rates of PJI.

Methods

A systematic review was performed using the electronic databases MEDLINE, Embase, and Web of Science. All records were screened in duplicate. Data collected included basic study characteristics, the details of the intervention and comparison solutions, if applicable, and rates of superficial and deep infection. A meta-analysis of comparative studies was performed to assess for consistency and potential direction of effect.

Results

A total of ten studies were included, of which one was an RCT, eight were retrospective cohorts, and one was a case series. In total, there were 29,630 TJAs in 29,596 patients. The mean age ranged from 61 to 80 years. Six studies compared povidone-iodine (Betadine®) to normal saline, two studies compared chlorhexidine to saline, one study compared “triple prophylaxis” to standard practice, and one study used gentamicin but had no comparison group. The pooled risk ratio for deep infection in studies using Betadine® compared to saline was 0.62 (95% confidence interval [CI]: 0.33-1.19), while for chlorhexidine it was 0.74 (95%CI: 0.33-1.65).

Discussion

Current evidence on the relative efficacy of irrigating solutions as prophylaxis for infection following TJA remains inconclusive. Imprecision of estimates vindicates the need for a definitive trial to further inform their use in surgical practice.

Conclusion

Antiseptic irrigation during TJA with solutions (Betadine®, chlorhexidine) may decrease PJI risk in patients undergoing primary and revision total hip and knee arthroplasties. Wide confidence intervals and heterogeneity among studies, however, render conclusions untrustworthy. Well-conducted RCTs are very much needed to help further investigate this issue.

## Introduction

Periprosthetic joint infection (PJI) following total joint arthroplasty (TJA) continues to be a devastating complication representing a significant health care expenditure [1-2]. Despite advances in sterility, antibiotic use, surgical techniques, and post-operative care, infection rates after TJA over the last 15 years have not decreased and may in fact be increasing [3]. The rates of TJA are rising globally, and by 2040, it is projected that over 6 million total knee and 2 million total hip arthroplasties will be performed in the United States alone [4]. Given the global projections, the identification of cost-effective interventions to reduce the risk of PJI following TJA remains critical.

The reduction of infection risk has focused on numerous patient factors which include optimizing obesity, diabetes, malnutrition, and smoking status in addition to surgical factors such as prophylactic and local antibiotics, skin preparation, operating room environment, duration of surgery, antibiotic cement, and wound irrigation [5-6]. Irrigation fluid is a potentially highly cost-effective “frugal innovation” target for the prevention of PJI. Irrigation minimizes bacterial contamination and is often delivered as normal saline alone, or with added concentrations of chlorhexidine or povidone-iodine (Betadine®) [7-9]. Both chlorhexidine and Betadine® have bactericidal effects and at appropriate dilution appear safe in terms of wound and tendon healing [7-10].

Evidence favoring irrigating solutions for the prevention of infection has been well studied in trauma [11]. Large trials have established standards for irrigating pressures and solutions. As important, evidence has furthered identified harms associated with previously popular solutions such as Castile soap. While this work in trauma may be generalizable to elective TJA surgery, important differences in the type of contamination, organisms present, and integrity of the soft tissues suggest cautious extrapolation, if any. 

We aimed to review the best available evidence evaluating irrigating solutions and irrigation pressures during TJA on the prevention of infection.

## Materials and methods

Protocol

The study protocol was designed a priori. The study was performed according to the Cochrane Handbook for Conducting Systematic Reviews. The manuscript is reported in adherence with the Preferred Reporting Items for Systematic Reviews and Meta-Analyses (PRISMA) [12].

Eligibility criteria

Eligibility criteria were established a priori. The inclusion criteria were as follows: 1) clinical studies, 2) patients undergoing primary or revision total hip or knee arthroplasty, 3) studies assessing various irrigation solutions or irrigation solutions delivered at different pressures, 4) studies comparing these solutions to a control solution or pressure, 5) reporting deep and/or superficial infection rate as outcomes, and 6) accessible in full text in English. Exclusion criteria were: 1) studies that primarily included patients who were undergoing surgery for a pre-existing infection, 2) hemiarthroplasties, unicompartmental arthroplasties, or resurfacings, and 3) overlapping reports of the same cohort (study with largest sample size included). Hemiarthroplasties, unicompartmental arthroplasties, and resurfacings were excluded as they each occur in specific populations that are not the same as the general TJA population, and thus carry varying levels of baseline risk. Abstracts were included only if they contained sufficient data on deep infection rates in each group.

Information sources

A search strategy was developed and executed though Web of Science, Embase, and MEDLINE. No date limits were used, and the search was performed on Jan 16, 2019.

Search

The search strategy included key terms relevant to arthroplasty, infection, and irrigation (see Appendices).

Study Selection

Following duplicate removal, all remaining records were exported to Rayyan (Qatar Computing Research Institute, Doha, Qatar). Rayyan is a free web-based software designed specifically for blinded review of articles. At least two reviewers (SE, TW, RM) independently screened each record. Any discrepancies at the title and abstract stages were resolved by automatic inclusion. At the full-text stage, discrepancies were resolved by consensus. For each stage of screening, inter-rater agreement was calculated using Cohen’s kappa (*k*) and classified a priori as follows: *k*<0.41: poor agreement, 0.41<*k*<0.60: moderate agreement, 0.61<*k*<0.80: substantial agreement, and *k*>0.80: excellent agreement [13].

Data Collection Process

Data was extracted using Google Sheets, an online spreadsheet (Google LLC, Mountain View, California, United States). The data extraction form was piloted prior to its implementation.

Data Items

Data on study design, location, patient demographics, follow-up, interventions and comparators, and outcomes were collected. The primary outcomes of interest were rates of deep infection. Secondary outcomes were rates of superficial infection and adverse events.

Risk of Bias in Individual Studies

Risk of bias was assessed using the Methodological Index for Non-Randomized Studies (MINORS) criteria for any non-randomized studies and the Cochrane Risk of Bias Tool 2.0 for any RCTs [14-15]. MINORS score is represented as median and range. Though no empirically established cut-offs exist for categorizing MINORS scores, the following thresholds were used based on previous literature: 0 < MINORS score < 6 = very low, 6 ≤ MINORS score < 10 = low, 10 ≤ MINORS score ≤ 14 = fair, and MINORS score > 14 = good [16].

Summary Measures

Information such as demographics is presented using descriptive statistics, with mean ± standard deviation (SD) or median ± interquartile range (IQR). Dichotomous outcomes are presented as risk ratio (RR) with 95% confidence intervals (CIs). All RRs are presented as intervention:control, such that RRs less than 1.0 favor intervention. Relative risk reductions (RRR) are also presented as needed for ease of interpretation.

Synthesis of Results

Demographic data is reported as a pooled weighted mean ± range. A meta-analysis was performed. It was determined a priori that a fixed effects (FE) model would be used if heterogeneity was < 20%; otherwise, a random-effects (RE) model would be utilized. When heterogeneity > or = 20%, we explored the sources for this. We hypothesized that potential sources of heterogeneity may include the intervention (i.e. the solution used), study design, and the presence of co-interventions.

## Results

Study selection

**Figure 1:**
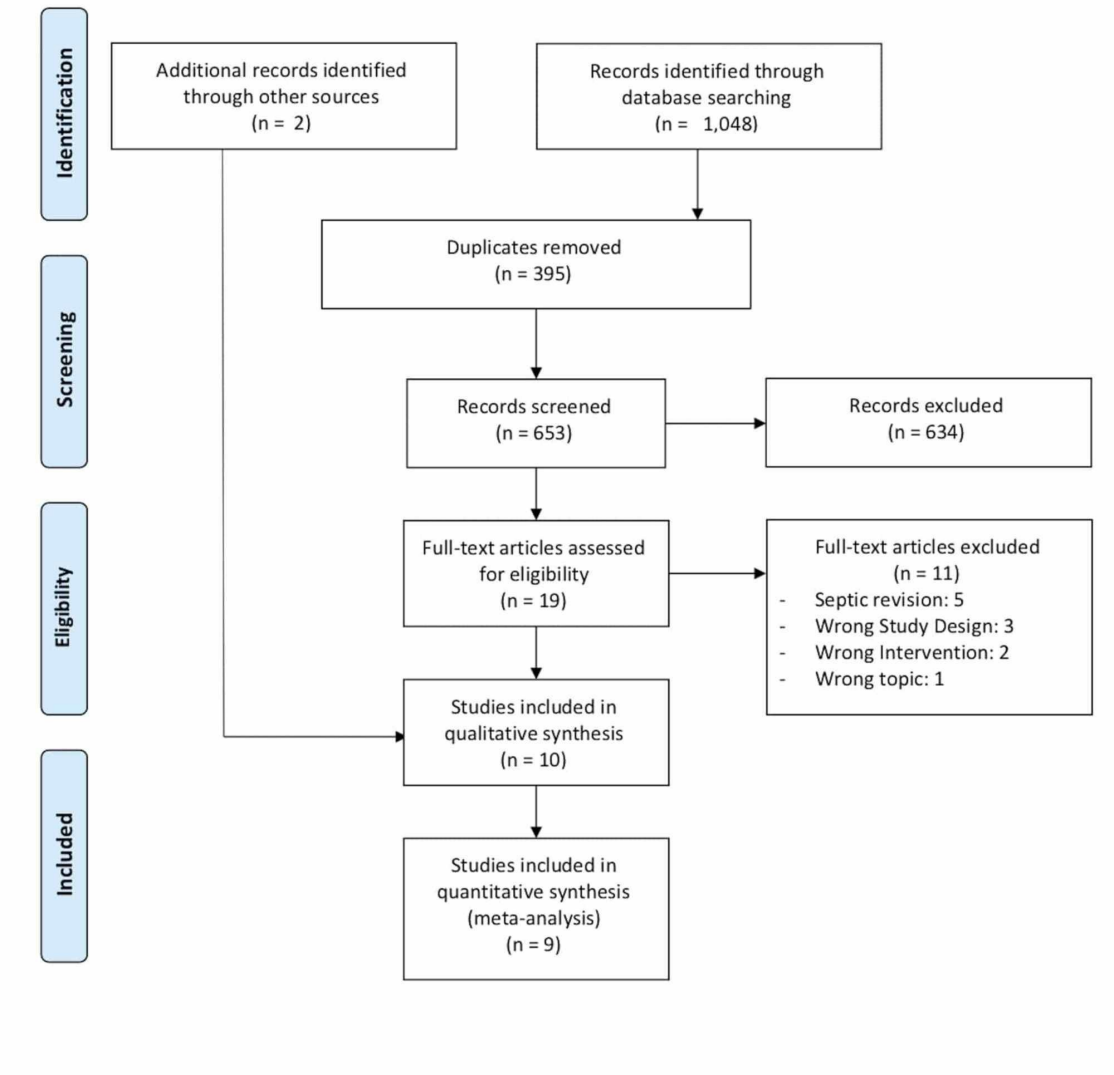
PRISMA flow diagram

The initial search returned 1048 results, from which 395 duplicates were removed. Ultimately, eight studies were selected for inclusion in the qualitative synthesis [8-10,17-21]. Two further studies were found by a manual search for a total of 10 studies [7,22]. There was moderate agreement at the title/abstract (*k* = 0.59, 95% CI: 0.38-0.80) and full text screening stages (*k* = 0.90, 95% CI: 0.71-1.00). Figure 1 shows the PRISMA flow diagram.

Study characteristics

Among the included studies, one study was an RCT, while the remaining studies were either retrospective cohorts (eight studies) or a case series (one study) [7-10,17-22]. Most studies were published between 2012 and 2019, with one study published in 1990. Seven studies focused on primary TJA surgeries and two studies on revision TJA [7-10,17-21]. One study included all of the above populations without subgroup data presented [20]. In total, there were 29,630 TJAs in 29,596 patients. Among studies separately reporting data for each joint, there were 14,183 hips and 12,168 knees. The mean age ranged from 61 to 80. Table 1 contains the characteristics of included studies.

**Table 1:** Characteristics of included studies NR, not reported [7-10], [17-22]

Author	Year	Patients	Joints	Minimum follow-up
Bortnem, et al. (1990)	1990	78	100	NR
Brown, et al. (2012)	2012	162	178	90 days
Calkins, et al. (2019)	2019	478	478	1 year
Frisch, et al. (2017)	2017	906	NR	1 year
Hart, et al. (2019)	2019	41	37	90 days
Hernandez, et al. (2019)	2019	11,738	11,738	90 days
Hofmann, et al. (2016)	2016	1,034	1,034	1 year
Rutgers, et al. (2018)	2018	4,494	4,494	1 year
Slullitel, et al. (2019)	2019	8,478	8,478	NR
Winkler, et al. (2018)	2018	744	744	6 months

Interventions and comparators

Six studies analyzed the use of a povidone-iodine (commonly known as Betadine®) solution compared to a control and two others compared chlorhexidine to control [7-9,16-17,21]. One study looked at the use of “triple prophylaxis”, which included povidone-iodine as the irrigation component (in addition to pre-operative vancomycin and nasal mupirocin) [19]. Table 2 summarizes the irrigation protocols for each study.

**Table 2:** Interventions and comparators across included studies PI, povidone-iodine; NR, not reported [7-10], [18-22]

Study	Intervention Solution	Solution Details	Delivery Method	Comparator
Bortnem, et al. (1990)	Gentamicin	NR	NR	N/A
Brown, et al. (2012)	Povidone-Iodine	0.35% solution (17.5 mL of 10% PI mixed with 500 mL of sterile saline)	Three-minute soak, followed by 1L pulsatile lavage with normal saline	Normal Saline
Calkins, et al. (2019)	Povidone-Iodine	0.35% solution (17.5 mL of 10% PI mixed with 500 mL of sterile saline)	Three-minute soak, followed by 1L pulsatile lavage with normal saline	Normal Saline
Frisch, et al. (2017)	Chlorhexidine	450 mL of 0.05% chlorhexidine gluconate solution	One-minute soak using Irrisept jet lavage device	Normal Saline
Hart, et al. (2019)	Povidone-Iodine	1 L of sterile 0.25% PI solution	Poured in three-minute soak, followed by saline irrigation	Normal Saline
Hernandez, et al. (2019)	Povidone-Iodine	1 L of sterile 0.25% PI solution	Poured in three-minute soak	Normal Saline
Hofmann, et al. (2016)	“Triple prophylaxis”: Povidone-Iodine, local antibiotic powder, intravenous antibiotics	0.1% solution (10 mL of 10% PI mixed with 1000 mL of sterile saline)	Poured in two-minute soak, followed by pulsatile saline lavage	Normal Saline
Rutgers, et al. (2018)	Chlorhexidine	NR	NR	Normal Saline
Slullitel, et al. (2019)	Povidone-Iodine	Various: 115 mL of non-sterile 10% PI in 500 mL of saline; 22.5 mL of sterile 10% PI in 250-500mL (0.2% to 0.35%)	One to three-minute soak, followed by saline lavage	Normal Saline
Winkler, et al. (2018)	Povidone-Iodine	15 mL of PI in 1-L normal saline (concentration not specified)	Bulb syringe for primary, pulsatile lavage for revision	Normal Saline

Risk of bias within included studies

The only RCT included in this review was at a low risk of bias for all domains [7]. The median MINORS score for seven comparative studies was 17/24 (range: 17-18) and for the one non-comparative study it was 7/16 [8-10,17-18,20-22]. Thus, all studies with the exception of the non-comparative study were of “good quality”, and thus, relatively low risk of bias. One study was only available in abstract form and thus not eligible for risk of bias assessment [19]. Table 3 contains a detailed MINORS assessment.

**Table 3:** Methodological index for non-randomized studies (MINORS) assessment [8-10,17-22]

	Clearly stated aim	Inclusion of consecutive patients	Prospective collection of data	Endpoints appropriate for aim	Unbiased assessment of endpoints	Appropriate follw-up period	Lost to follow-up <5%	Prospective calculation of sample size	Adequate control group	Contemporary groups	Baseline equivalence of groups	Adequate statistical analysis	Total score
Brown, et al. (2012)	2	2	0	2	0	2	1	0	2	2	2	2	17
Bortnem, et al. (1990)	0	2	0	2	0	2	1	0					7
Frisch, et al. (2017)	2	2	0	2	0	2	1	0	2	2	2	2	17
Hart, et al. (2019)	2	2	0	2	0	2	1	0	2	2	2	2	17
Hernandez, et al. (2019)	2	2	0	2	0	2	1	0	2	2	2	2	17
Hofmann, et al. (2016)	2	2	0	2	0	2	1	0	2	2	2	2	17
Slullitel, et al. (2019)	2	2	0	2	0	2	2	0	2	1	1	2	16
Winkler, et al. (2018)	2	2	0	2	0	2	2	0	2	2	2	2	18

Synthesis of Results

Antiseptic Solutions

**Figure 2:**
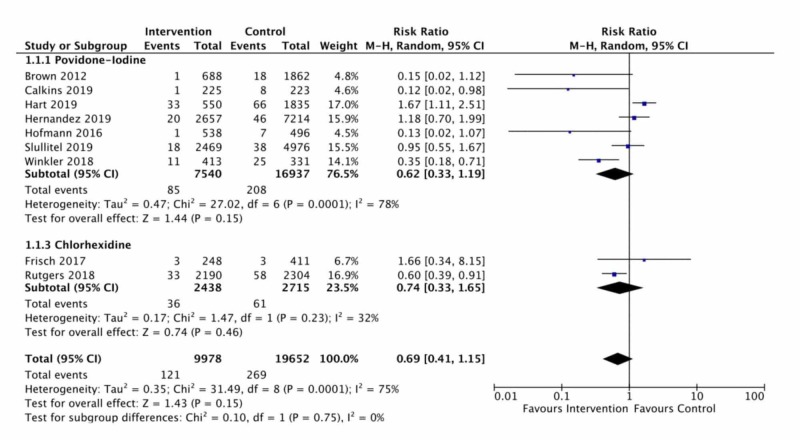
Forest plot of povidone-iodine and chlorhexidine effects and pooled effect CI. confidence interval [7-10], [17-18], [19-20,22]

In a pooled analysis (nine studies, 29,630 patients), antiseptic solutions, although suggestive of an important reduction, did not significantly reduce the risk of infection following TJA (RR=0.69, 95%CI: 0.41-1.15, I2 = 75%). Heterogeneity in this pooled estimate vindicated exploration for potential a priori factors including type of irrigating solution and study design. Given that all but one study was a retrospective cohort, and that all comparative studies had similar risk of bias, this was not seen as a necessary subgroup analysis. On the other hand, the different irrigation solutions were analyzed as subgroups (see below). Figure 2 displays the forest plot for this meta-analysis.

Type of Solution

Betadine® irrigation (seven studies, 24,477 patients), although suggestive of an important reduction, did not significantly reduce risk of deep infection compared to normal saline (RR = 0.62, 95%CI: 0.33-1.19, I2 = 78%). Similarly, chlorhexidine irrigation (two studies, 5,153 patients), although suggestive of a reduction, did not significantly reduce infection risk compared to normal saline (RR=0.74, 95%CI: 0.33-1.65, I2 = 32%). A single retrospective case series assessed the impact of gentamicin lavage on 100 arthroplasty procedures, including revision and fracture patients. Overall, three patients (3%) had wound drainage, and two patients (2%) developed a deep infection requiring revision surgery. No comparison group was available [21].

Study Designs

The only RCT evaluating the effectiveness of povidone-iodine (PI) lavage found that within 90 days, the rate of PJI in the intervention group was 0.4% compared to 3.5% in the control group (RR: 0.12, 95%CI: 0.02 to 0.98, *p* = 0.037). There were no significant differences between the two groups in terms of other major wound complications [7]. Similarly, a retrospective cohort study of 2540 primary TJAs found an infection rate of 0.15% among those who received the same PI lavage as above, compared to 0.97% in the pre-PI cohort (RR: 0.15, *p* = 0.04 [17]. Another retrospective cohort looking at dilute PI irrigation and vancomycin powder found a decrease in infection rates across all primary and revision TJA (Odds Ratio (OR) range: 0.21-0.47), though the effect was only significant for primary total knee arthroplasty (TKA) [18].

On the other hand, two large studies, both performed at the same centre, assessed the same PI lavage solution (0.25% PI in 1L NS for three minutes) in primary and revision [8-9] TJA. Neither study found a significant difference in infection rates between the pre- and post-PI lavage cohorts. Furthermore, a large retrospective cohort study which included primary TJAs found a deep infection rate of 0.8% in the PI group compared 0.9% in the control group (*p* = 0.762) [22]. Finally, one study which looked at “triple prophylaxis” including dilute betadine lavage in primary TJA found a significant reduction in the rate of deep (1.4% vs. 0.2%, *p* = 0.02) but not superficial (0.6% vs. 0.6%, *p* = 0.92) infection [20].

Two retrospective cohort studies have assessed the rates of infection before and after the establishment of a dilute chlorhexidine lavage protocol [10,19]. Only one study reported the exact solution, which consisted of NS and 0.05% chlorhexidine gluconate solution prior to closure [10]. One study found a significantly lower (1.5% vs. 2.5%) rate of deep infection in the chlorhexidine group compared to control (RR: 0.60, 95%CI: 0.39-0.91, *p* = 0.009) [19]. The other study found no difference in the rate of deep (0.7% control vs. 1.2% chlorhexidine, RR: 1.7, 95% CI: 0.3-8.2, *p* = 0.53) or superficial (0.7% vs. 0.8%, OR: 0.9, 95%CI: 0.2-5.4, *p* = 0.913) infection between the two groups [10].

Irrigating Pressures

No studies specifically assessing the impact of various irrigation pressures were identified. Five of the included studies specified how the irrigation fluids were delivered to the wound [8-10,18,20]. Among these studies, three poured in a povidone-iodine solution, one used a purpose-made jet lavage device (Irrisept®) to deliver chlorhexidine solution, and one study using povidone-iodine utilized a bulb syringe for primary cases and a pulsatile lavage for revision cases.

Sensitivity Analysis

**Figure 3:**
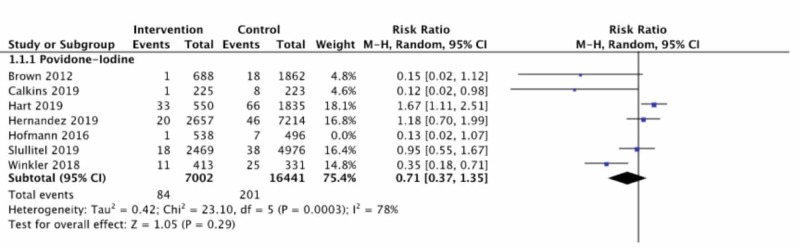
Sensitivity analysis for povidone-iodine with one study which used local antibiotic powder removed CI. confidence interval [7-9], [17-18], [20], [22]

Given that one study used PI as well as a local antibiotic powder (i.e. “triple prophylaxis” as termed in that study) [20], a post-hoc sensitivity analysis was performed by removing that study from the analysis for potential confounding effect. This slightly reduced the RRR for PI to 29% (RR 0.71, 95%CI: 0.37 to 1.35), suggesting that local antibiotic may have only a small effect on the overall estimate of effect for PI (Figure 3). 

## Discussion

In a systematic review and meta-analysis of irrigating solutions in TJA, we found point estimates of effect favoring irrigating solutions (Betadine®, Chlorhexidine) versus normal saline in the reduction of infection. However, wide confidence intervals and between-study heterogeneity limit the validity of our findings. Between-study variations in effect may be explained by study quality and type of irrigating solutions. Six studies evaluated povidone-iodine, of which 4 showed significant improvements in the rates of PJI. The largest RCT to date on this topic demonstrated an 88% RRR associated with the use of Betadine® lavage. The pooled data for all Betadine® studies revealed a 39% RRR.

The strengths of this study include its rigorous methodology with an a priori protocol, duplicate screening and data extraction, broad search strategy, and the inclusion of a large total sample size. Our findings are limited by between-study heterogeneity. We explored potential causes of study differences by study design, irrigating fluid type, formulation, and surgical protocols. Our findings suggested that there is an important, though imprecisely estimated, effect for both chlorhexidine and Betadine® in the context of primary and revision TJA surgery.

Povidone-iodine has been shown to be an optimal irrigation fluid in the intraoperative setting when compared to other solutions in vitro. Furthermore, at the minimal bactericidal concentration, Betadine® was shown to be the least cytotoxic [23]. Positive results have also been shown in spine surgery, where Cheng *et al*. compared a 0.35% Betadine® irrigation solution to normal saline in an RCT of 414 patients. They showed a significantly decreased rate of deep and total infection in the Betadine® group (*p *= 0.0146 and *p *= 0.0072, respectively) [24]. Similarly, Chang *et al*. in 2006 showed out of 244 consecutive patients undergoing lumbosacral posterolateral fusions, patients who had 0.35% Betadine® irrigation had lower postoperative infection rates compared to saline (*p *< 0.05) [25]. It is difficult to assess what the optimal dosing of Betadine® is with the included studies ranging from 0.1% to 0.45% [8-9,17,20,22] and some not reporting the concentration [7,18]. In an in vitro model assessing minimal bactericidal concentration and cytotoxicity of cells, the optimal dosing was found to be 1.3g/L [23].

Two studies evaluated the use of chlorhexidine in the prevention of PJI [10,19]. Chlorhexidine demonstrated a RRR of 26%, again showing improvement in the rates of PJI compared to the control group. In general, chlorhexidine is bactericidal and works by disruption of the cell membrane [10]. In animal studies, it has been shown to be safe in terms of its effects on wound healing, tendon properties, and collagen [26- 27]. Furthermore, it has been shown to be more efficacious in removing methicillin-resistant *Staph. aureus* (MRSA) biofilms than other solutions [28]. In this study, only 2438 patients had chlorhexidine as the irrigation fluid as compared to 5071 patients who had Betadine® when pooling the included studies. Thus, there is less certainty in the results of chlorhexidine studies compared to Betadine®.

No included studies directly assessed the role of various irrigating pressures. A large international trauma RCT found no significant difference between high, low, and very low pressures in open fractures [11]. On the other hand, an RCT of 356 patients with hip fractures undergoing hemiarthroplasty found that those irrigated with pulsatile lavage had significantly lower infection rates compared to gravity flow (saline used in both groups, RR: 0.35, 95%CI: 0.17 to 0.72) [29]. The studies included in this review employed a range of irrigation strategies, including bulb syringe, pulsed lavage, and gravity flow, though none of the studies actively compared two different pressure protocols. Thus, it remains unclear which of pulsed lavage, bulb syringe, or gravity flow provides the optimal irrigation pressure in the context of TJA surgery.

Despite the fact that there is a recently published RCT of over 400 patients, that study demonstrated a high degree of imprecision with large confidence intervals (RR: 0.12, 95%CI: 0.02 to 0.98). Furthermore, that study included revision surgeries only, which account for less than 10% of the overall TJA volume [30]. Currently, evidence on the appropriate irrigating solution or irrigation pressure during arthroplasty of the hip and knee remains inconclusive. Given the widespread use of each approach and the potential for important benefits, or possible harms, definitive studies are needed. This research will inevitably require significantly larger samples of patients to have enough precision to determine effectiveness in infection reduction in both primary and revision procedures.

## Conclusions

The use of antiseptic irrigation solutions (Betadine®, chlorhexidine) appears to decrease PJI in patients undergoing primary and revision total hip and knee arthroplasties. Both solutions seem to have similar effects on the reduction of infection rates. Despite this, the overall quality of available studies remains low, and estimates of effect are associated with considerable uncertainty. This is an emerging area of study, and large RCTs are needed to answer these questions definitively. In the meantime, it is difficult to recommend a specific solution in the context of primary TJA; however, given their well-established safety profile and the likely presence of a signal in favor of both Betadine® and chlorhexidine, it would not be unreasonable to use these solutions routinely at the surgeon's discretion.

## Appendices

**Table 4:** Full Search Strategy

	MEDLINE/Embase	Web of Science/CENTRAL
Search strategy	exp Infection/ or exp Surgical Wound Infection/ or infection.mp. exp Arthroplasty, Replacement, Knee/ or exp Arthroplasty/ or exp Arthroplasty, Replacement, Hip/ or Arthroplasty.mp. irrigation.mp. exp Anti-Infective Agents, Local/ or wound irrigation.mp. or exp Anti-Bacterial Agents/ 3 or 4 1 and 2 and 5 Limit 6 to humans	Irrigation or Lavage Total joint replacement or Total joint arthroplasty or Total hip replacement or Total hip arthroplasty or Total knee replacement or Total knee arthroplasty Infection or septic arthritis or septic joint or periprosthetic joint infection 1 and 2 and 3
Number of papers retrieved	MEDLINE: 215 Embase: 744	Web of Science: 324 CENTRAL: 186

## References

[1] Kapadia BH, McElroy MJ, Issa K, Johnson AJ, Bozic KJ, Mont MA (2014). The economic impact of periprosthetic infections following total knee arthroplasty at a specialized tertiary-care center. J Arthroplasty.

[2] Kapadia BH, Banerjee S, Cherian JJ, Bozic KJ, Mont MA (2016). The economic impact of periprosthetic infections after total hip arthroplasty at a specialized tertiary-care center. J Arthroplasty.

[3] Bozzo A, Madden K, Pond G (2019). Risk factors for prosthetic joint infection following primary total hip arthroplasty: a 15-year population-based cohort study. J Bone Joint Surg Am.

[4] Singh JA, Yu S, Chen L, Cleveland JD (2019). Rates of total joint replacement in the United States: future projections to 2020-2040 using the national inpatient sample. J Rheumatol.

[5] Kassim RA, Saleh KJ, Yoon P, George SM, Greg B, Steven H (2012). Varus distal femoral osteotomy. Tech Knee Surg.

[6] Parvizi J, Saleh K, Mont M (2008). Efficacy of antibiotic-impregnated cement in total hip replacement. Acta Orthopaedica.

[7] Calkins TE, Culvern C, Nam D (2020). Dilute Betadine lavage reduces the risk of acute postoperative periprosthetic joint infection in aseptic revision total knee and hip arthroplasty: a randomized controlled trial. J Arthroplasty.

[8] Hernandez NM, Hart A, Taunton MJ (2019). Use of povidone-iodine irrigation prior to wound closure in primary total hip and knee arthroplasty. J Bone Joint Surg Am.

[9] Hart A, Hernandez NM, Abdel MP (2019). Povidone-iodine wound lavage to prevent infection after revision total hip and knee arthroplasty. J Bone Joint Surg.

[10] Frisch NB, Kadri OM, Tenbrunsel T, Abdul-Hak A, Qatu M, Davis JJ (2017). Intraoperative chlorhexidine irrigation to prevent infection in total hip and knee arthroplasty. Arthroplast Today.

[11] FLOW Investigators, Petrisor B, Sun X (2011). Fluid lavage of open wounds (FLOW): a multicenter, blinded, factorial pilot trial comparing alternative irrigating solutions and pressures in patients with open fractures. J Trauma.

[12] Moher D, Liberati A, Tetzlaff J, Altman DG, PRISMA Group (2009). Preferred reporting items for systematic reviews and meta-analyses: the PRISMA statement. PLoS Med.

[13] Landis JR, Koch GG (1977). The measurement of observer agreement for categorical data. Biometrics.

[14] Slim K, Nini E, Forestier D, Kwiatkowski F, Panis Y, Chipponi J (2003). Methodological index for non-randomized studies (MINORS): development and validation of a new instrument. ANZ J Surg.

[15] Higgins JPT, Sterne JAC, Savovic J (2016). A revised tool for assessing risk of bias in randomized trials. Cochrane Database of Systematic Reviews 2016.

[16] Ekhtiari S, Horner NS, Bedi A, Olufemi RA, Khan M (2018). The learning curve for the Latarjet procedure: a systematic review. Orthop J Sports Med.

[17] Brown NM, Cipriano CA, Moric M, Sporer SM, Della Valle CJ (2012). Dilute Betadine lavage before closure for the prevention of acute postoperative deep periprosthetic joint infection. J Arthroplasty.

[18] Winkler C, Dennison J, Wooldridge A (2018). Do local antibiotics reduce periprosthetic joint infections? A retrospective review of 744 cases. J Clin Orthop Trauma.

[19] Rutgers M, Yesilkaya F, Moojen D (2018). Reduction in prosthetic joint Infection after introduction of per-operative wound irrigation with chlorhexidine: A large scale evaluation of a change in standard care. 13th Congress of the European Hip Society.

[20] Hofmann KJ, Hayden BL, Kong Q, Pevear M, Cassidy C, Smith E (2017). Triple prophylaxis for the prevention of surgical site infections in total joint arthroplasty. Curr Orthop Pract.

[21] Bortnem KD, Wetmore RW, Blackburn GW, Brownell SM, Page BJ 2nd (1990). Analysis of therapeutic efficacy, cost, and safety of gentamicin lavage solution in orthopaedic surgery prophylaxis. Orthop Rev.

[22] Slullitel P, Dobransky J, Bali K (2019). Is there a role for preclosure dilute Betadine irrigation in the prevention of postoperative infection following total joint arthroplasty?. J Arthroplasty.

[23] Van Meurs SJ, Gawlitta D, Heemstra KA, Poolman RW, Vogely HC, Kruyt MC (2014). Selection of an optimal antiseptic solution for intraoperative irrigation: an in vitro study. J Bone Joint Surg Am.

[24] Cheng M Te, Chang MC, Wang ST, Yu WK, Liu CL, Chen TH (2005). Efficacy of dilute betadine solution irrigation in the prevention of postoperative infection of spinal surgery. Spine (Phila Pa 1976).

[25] Chang FY, Chang MC, Wang ST (2006). Can povidone-iodine solution be used safely in a spinal surgery?. Eur Spine J.

[26] Brennan SS, Foster ME, Leaper DJ (1986). Antiseptic toxicity in wounds healing by secondary intention. J Hosp Infect.

[27] Han Y, Giannitsios D, Duke K (2011). Biomechanical analysis of chlorhexidine power irrigation to disinfect contaminated anterior cruciate ligament grafts. Am J Sports Med.

[28] Smith DC, Maiman R, Schwechter EM, Kim SJ, Hirsh DM (2015). Optimal irrigation and debridement of infected total joint implants with chlorhexidine gluconate. J Arthroplasty.

[29] Hargrove R, Ridgeway S, Russell R, Norris M, Packham I, Levy B (2006). Does pulse lavage reduce hip hemiarthroplasty infection rates?. J Hosp Infect.

[30] (2020). Hip and Knee Replacements in Canada, 2017-2018: Canadian Joint Replacement Registry Annual Report. Ottawa, ON. Epub ahead of print.

